# Behavioral Components and Their Tailoring in Participatory Health Interventions for Precision Prevention

**DOI:** 10.1055/s-0044-1800715

**Published:** 2025-04-08

**Authors:** Kerstin Denecke, Octavio Rivera Romero, Carlos Luis Sanchez Bocanegra, Talya Miron-Shatz, Rolf Wynn, WG contribution to the IMIA Yearbook 2023

**Affiliations:** 1 Bern University of Applied Sciences, Bern, Switzerland; 2 Department of Electronic Technology, Universidad de Sevilla, Sevilla, Spain; 3 Faculty of Health Sciences, Universitat Oberta de Catalunya (UOC), Spain; 4 Faculty of Business Administration, Ono Academic College, Kiryat Ono, Israel; 5 Department of Clinical Medicine, UiT The Arctic University of Norway, Tromsø, Norway; 6 Department of Education, ICT and Learning, Østfold University College, Halden, Norway

**Keywords:** Participatory health informatics, behavior change, precision prevention, digital health intervention

## Abstract

**Objective** : To study which behavioral components are implemented within participatory health interventions for precision prevention, specifically how they are realized as part of the interventions and how the tailoring of the interventions is implemented.

**Methods** : We selected three case studies of participatory health interventions for precision prevention for three different target groups (children, parents, older adults with chronic conditions). One author with a background in psychology mapped the interventions and the digital functionalities to the 9 intervention functions of the behavioral change wheel (education, persuasion, incentivisation, coercion, training, enablement, modeling, environmental restructuring, restrictions).

**Results** : While the intervention functions persuasion, incentivisation, education, modeling and coercion are implemented in all three interventions under considerations, two techniques (restrictions, and environmental restructuring) were not implemented in any of the three solutions. Training was only applied in one application and enablement in two interventions. We identified significant evidence gaps in both the tailoring process and the effectiveness of behavior change techniques in precision prevention.

**Conclusion** : We conclude that there is a need for more focused studies on the effects of behavior interventions functions in digital health interventions and for design guidelines to improve these interventions for personalized health outcomes, thereby advancing precision prevention in digital health.

## 1. Introduction

The pursuit of precision prevention in public health has gained considerable attention and prominence in recent years [ 1 ]. This paradigm shift places emphasis on tailoring health interventions to individual characteristics and needs, with the overall aim of increasing the effectiveness of preventive interventions [ 2 ]. In this context, participatory health interventions have emerged as a dynamic approach that promotes the active involvement of individuals and communities in the design and implementation of prevention strategies [ 3 ]. We define ‚prevention‘ as encompassing interventions aimed at averting potential health conditions or diseases, as well as promoting healthy lifestyles. Precision means tailoring prevention strategies to individual characteristics, risks and circumstances, rather than taking a one-size-fits-all approach.

This definition sets the context for our exploration of behavior components in participatory health interventions for precision prevention since the success of participatory health interventions for prevention depends on a thorough understanding of the complex interplay of behavioral components and their tailoring for the target population [ 4 ]. Without comprehending these change mechanisms, there is a constraint on advancing the development of successful behavior change interventions [ 5 ]. Behavioral components encompass a spectrum of factors, from individual choices and socio-cultural determinants to genetic predispositions, that collectively influence health-related behaviors.

A behavior change technique is a methodological procedure that is an integral part of interventions designed to change specific behaviors [ 6 ]. This structured approach defines these techniques as critical elements in the effectiveness of behavior change interventions. These techniques are theory-based and are used to change one or more determinants of behavior, such as a person's attitude or self-efficacy [ 7 ]. Consequently, unraveling and harnessing these behavioral components has become paramount in the pursuit of precision prevention strategies that can address the unique needs and circumstances of diverse populations. Mair et al. tried to identify in their review the most effective behavior change techniques in digital health interventions that address the prevention or management of non-communicable diseases [ 8 ]. They found evidence that “credible source, social support, prompts and cues, graded tasks, goals and planning, feedback and monitoring, human coaching and personalization components increase the effectiveness of digital health interventions targeting the prevention and management of these diseases”.

The aim of this work is to study how behavioral components are used within participatory health interventions for precision prevention and the role of the digital solution in tailoring the intervention. In contrast to the work of Mair et al. we want to find out whether there are differences in designing participatory health interventions addressing different target groups, what are best practices and which limitations exist.

## 2. Method

We selected three case studies related to participatory health interventions for precision prevention. One author with a background in psychology mapped the interventions to the nine intervention functions (education, persuasion, incentivisation, cercision, training, enablement, modeling, environmental restructuring, restrictions) of the behavior change wheel (BCW). The BCW integrates different frameworks for categorizing behavior change interventions. It also provides a clear structure that facilitates the categorization of intervention functions [ 9 ]. As Michie *et al.* , point out, the BCW offers a “structured methodology for relevant intervention functions and policy categories based on what is understood about the target behavior” [ 9 ].

The intervention functions of the BCW encompass a range of strategies to facilitate behavior change:

- Education: Increases knowledge or understanding, like providing information to promote healthy eating;
- Persuasion: Uses communication to induce feelings or stimulate action, such as using imagery to motivate physical activity;
- Incentivisation: Creates an expectation of reward, for instance, using prize draws to encourage smoking cessation;
- Coercion: Involves creating an expectation of punishment or cost, like raising the financial cost to reduce excessive alcohol consumption;
- Training: Imparts skills, as seen in advanced driver training to promote safe driving;
- Restriction: Utilizes rules to reduce the opportunity to engage in the target behavior, such as prohibiting sales of certain substances to minors;
- Environmental restructuring: Changes the physical or social context, for example, providing on-screen prompts for general practitioners to ask about smoking behavior;
- Modeling: Provides an example for people to aspire to or imitate, like using TV drama scenes to increase safe-sex practices;
- Enablement: Increases means or reduces barriers to enhance capability (beyond education and training) or opportunity (beyond environmental restructuring), such as providing behavioral support for smoking cessation or medication for cognitive deficits.

Each of these functions targets different aspects of behavior change, offering a comprehensive framework for designing effective health interventions. For the intervention functions, we studied how this is realized from a technical perspective in participatory health interventions aiming at precision prevention.

To identify case studies, we conducted searches in PubMed with keywords related to:

- Field of medicine: Prevention and preventive medicine;
- Medical condition: Smoking cessation, healthy eating, physical activity, alcohol abuse, unhealthy behavior;
- Device: mHealth, wearable, smartphone, digital health;
- Study design: Experimental study, randomized control trial;
- Target population: children, elderly, silver age, old adults.

The search was conducted on 27 ^th^ September 2023. The search resulted in 134 matches with 86 papers published in the last five years. We manually selected three case studies focusing on different target populations, namely children [ 10 ], older adults with chronic conditions [ 11 ], and parents [ 12 ]. In addition, the three studies focus on a mix of healthy behaviors, using different technologies (web and app) and different strategies (individual, community, and proxy). Two focus on obesity prevention, and the third on lifestyle management for people with chronic conditions. Although they do not cover all possible behaviors, these studies have evaluated the effectiveness of their interventions, at least through pre-post evaluations.

## 3. Results

In this section, we summarize the selected case studies. For each paper, we identified the BCW intervention functions and identified how the function was realized in the participatory health intervention. Additionally, we describe the role of the used digital solution to tailor the intervention in each study case.

### 3.1. Case studies

The three studies used different intervention types: Internet-based [ 10 ], mobile health [ 12 ], and a community-based e-health intervention [ 11 ]. A digital solution was specifically designed to deliver the intervention in two of the three cases [ 10 , 11 ]. An already available school-communication app was used in the other case [ 12 ]. The studies dealt with obesity [ 10 ], unhealthy diet [ 12 ], and chronic disease management [ 11 ]. All the studies followed an experimental or quasi-experimental study design. All three studies found that technology-based interventions were effective in improving participants' health. The Thai [ 10 ] and Australian [ 12 ] studies evaluated interventions targeting school children, while the older adults study evaluated an intervention targeting older adults with chronic illnesses. The Thai and Australian studies lasted four and six weeks, respectively, while the older adults study lasted eight weeks [ 11 ]. Table 1 provides more details on the studies.

**Table 1.** Description of the three case studies.

Study aspect / Reference	Rerksuppaphol et al. [ 10 ]	Sutherland et al. [ 12 ]	Wu et al. [ 11 ]
Country	Thailand	Australia	Singapore
Intervention type and objective	Internet-based obesity prevention program	m-health intervention to improve the nutritional quality of lunch boxes	Community e-health program to improve chronic disease self-care and health literacy
Target population	School children	Parents of school children	Older adults with chronic diseases
Settings	Schools	Schools	Community
Duration	4 months	10 weeks	8 weeks
Study Design	Experimental study (2-arms RCT)	Experimental study (4-arms RCT)	Quasi-experimental study (2-groups Pre-post)
Measurements	Demographic and anthropometric characteristics	Energy content of food packed in children's lunch boxes. Intervention feasibility and acceptability	Self-care, healthy aging, health literacy, empowerment, social support, and health outcomes
Data collection	Baseline and monthly	Baseline and post	Baseline (pre) and post
Type of digital solution	Smartphone app	School-communication app	App (Care4Senior) + Web platform (used by researchers)
Digital solution was specifically designed for the intervention	Yes	No	Yes
Features implemented in the digital solution	- Data collection - Interpretation of nutritional status (normal, overweight, or obese) - Educational content and recommendations on healthy nutrition, food habits, and physical activity	- Notifications - Static content	Health education on the management of hypertension, hyperlipidemia, and diabetes, brain health, healthy diet, lifestyle modification, medication adherence, exercise, and mindfulness practice.
Target behavior/ condition	Obesity	Unhealthy diet	Chronic disease management
Results	Significant decrease in BMI, waist-hip ratio, and consumption of foods rich in saturated fats and sugary drinks	Significant increase in total nutritional quality score, consumption of recommended foods, and decrease in consumption of discretionary foods	Significant increase in chronic illness self-care and health literacy
Conclusions	The internet-based obesity prevention program was effective in reducing BMI, waist-to-hip ratio, and consumption of foods high in saturated fat and sugary drinks in Thai school children.	The m-health intervention „SWAP IT“ was effective in improving the nutritional quality of school children's lunch boxes, increasing the consumption of recommended foods, and reducing the consumption of discretionary foods. The intervention was also feasible and acceptable to parents.	The community-based e-health intervention for older adults with chronic conditions was effective in improving participants' chronic conditions, self-care, and health literacy.
Limitations	The study was of short duration and was carried out in a single country.		The study was a pilot with a small sample size.

### 3.2. Tailoring implemented in the digital solutions

Tailoring is a key component in precision prevention interventions. Although the three study cases reported a tailored intervention, this section focuses on the role of the used digital solution to tailor the intervention in each study case. In this regard, the digital solution used by Sutherland et al. [ 12 ] did not automatically perform any tailoring. Quantitative data manually entered into the digital solution by assistants or end users were used as the basis for tailoring in the other two case studies. Both digital solutions personalized their contents and functionalities based on the collected data and explicitly mentioned their theoretical foundations. Content was previously created by experts. Regarding the type of tailoring, Rerksuppaphol *et al.* , [ 10 ] used a dynamic approach in which tailoring was monthly performed once new collected data were entered. Wu *et al.* , [ 11 ] followed a mixed tailoring approach. Feedback, inter-human interaction, and user-targeting tailoring strategies were implemented in the digital solutions used in both cases. Adaptation was also implemented in by Rerksuppaphol *et al.* [ 10 ]. None of the studies reported the specific tailoring algorithm implemented in the digital solutions. Table 2 summarizes relevant tailoring aspects included in the two digital health interventions that integrated automatic tailoring.

**Table 2.** Description of the three case studies.

Tailoring aspects / References	Rerksuppaphol et al. [ 10 ]	Wu et al. [ 11 ]
Considered factors	Demographic and anthropometric data	Demographic and health data
Data collected	Age Gender Weight Height Waist circumference Hip circumference	Examples of health data explicitly mentioned: Glucose, Blood pressure
Tools and metrics	Electronic scale Height rod Non stretch tape	Self-reported questionnaires
Collection process	Trained research assistants collected data. They entered data into the digital solution (intervention group).	Questionnaires delivered in face-to-face meeting (baseline) and self-reported data
Tailored components [ 13 ]	- Content - Functionality	- Content - Functionality
Tailored component description	- Recommendations on healthy nutrition and physical activity based on the individual's nutritional status and age. Feedback on the nutritional status evolution.	Health education content Feedback on daily care
Foundations	Nutritional status based on the criteria defined by the WHO	Social Cognitive Theory
Tailoring type	Partially dynamic The digital solution provides tailored content based on the updated data	Mixed (monitoring was dynamic, but health education was static in the app)
Tailoring techniques [ 14 ]	- Adaptation - Feedback - Inter-Human Interaction - User targeting	- Feedback - Inter-Human Interaction - User targeting
Tailoring algorithm	Unknown	Unknown

### 3.3. Adopted BCW intervention functions

Table 3 shows which intervention functions were adopted to the digital health intervention. We can see that four [ 10 ], six [ 12 ], or seven [ 11 ] functions were reported. The functions persuasion, incentivisation, education, modeling and coercion were adopted in all three interventions under considerations.

**Table 3.** Intervention functions adopted in each of the reviewed cases (BCW - Behavior change wheel)

Used BCW Intervention components / References	Rerksuppaphol et al. [ 10 ]	Sutherland et al. [ 12 ]	Wu et al. [ 11 ]
Persuasion (stimulate action)	Through individual instructions given to students by the solution (intervention group) or by research assistants (control group).	Through in-app push-messages to parents	Through individual advice given to participants in face-to-face meetings Monitoring of activity by the digital solution
Incentivisation (expectations of reward)	Improved measurements: weight, hight, BMI, circumference	More healthy lunch-box for students Photos taken of lunch-boxes and evaluated by dietician	Improved blood-test results Improved results on questionnaires / psychometrics, etc.
Education (increase knowledge)	Education given face-to-face by teachers as well as in app	Education given face-to-face by teachers to students as well as in app/push-messages to parents	Education given face-to-face by instructors as well as in app
Coercion (expectations of punishment)	No improvement in measurements: weight, hight, BMI, circumference	Photos taken of lunch-boxes and evaluated by dietician	No improvement in blood-test results No improvement in results on questionnaires, psychometrics, etc.
Enablement (Increase capability)	N/A	Water bottle marked with ‘water only’ and cooling device for lunch box sent to families	Weekly meetings
Environmental restructuring (Change in physical environment)	N/A	N/A	N/A
Training (impart skills)	N/A	N/A	Exercises and quizzes implemented in the app
Restrictions (reduce opportunity to engage in behavior)	N/A	N/A	N/A
Modeling (examples to imitate)	Information about desirable weight/BMI given in app	Parents given examples of desirable lunch-boxes in app Children given examples in class	Participants shown videos
Total reported functions	4	6	7

Restrictions, and environmental restructuring was not implemented in any of the three solutions. Training was only applied in one application [ 11 ], and enablement in two interventions [ 11 , 12 ]. While all three interventions used persuasion in the intervention itself, *i.e.* , in a digital manner, education was delivered in a face-to-face way in all three interventions.

## 4. Discussion

In this paper, we investigated which behavioral components are adopted in participatory health interventions for precision prevention, and how tailoring is realized to achieve precision prevention. For this purpose, we analyzed three different case studies, each with a focus on a different condition (obesity, healthy eating, and chronic disease management). Furthermore, the target population was diverse (children, parents, the elderly). The studies were published in 2017, 2019, and 2022, allowing us to gain exemplary insights into precision prevention research over the past seven years and to draw conclusions about the limitations of research in this area.

Although a wide variety of digital solutions including gaming consoles, targeted computer and web-based programs, or social media have been used to implement prevention interventions [ 15 ], smartphone apps have gained an increasing interest in the research community mainly because of their penetration and their capabilities that enable the implementation of just-in-time interventions and ecological momentary assessments [ 16 ]. In our case studies both, general purpose and specifically designed apps were used to implement participatory interventions for precision prevention. For some interventions, it may be sufficient to implement them using available digital solutions, as was done in the study by Sutherland *et al.* , [ 12 ] who used an existing app. The advantage of such an approach is that the intervention is easier to develop, and the digital solution can be selected based on the previous experience of the target user group. In contrast, when digital solutions are specifically designed as in the other two studies [ 10 , 11 ], it is possible to implement specific features to realize behavioral components such as reminders, tracking or monitoring functionalities.

Only seven of the nine BCW intervention functions could be identified in the three case studies. Restrictions and environmental restructuring were not adopted. One reason may be that these techniques lack effectiveness for targeted prevention. For example, Martin *et al.* , [ 16 ] found no evidence for the effectiveness of environmental restructuring for the prevention of childhood obesity.

In our review of the three studies, we identified a significant gap: none of the studies provided details of the rationale for the intervention design choices, particularly regarding the inclusion or exclusion of specific features. This lack of transparency is concerning, as various factors such as the digital literacy of the target population, their cognitive abilities, and existing evidence on the efficiency and effectiveness of each intervention feature for precision prevention are critical in shaping these decisions. To improve the replicability and understanding of such studies, it is imperative that future research explicitly outlines the rationale behind design decisions. Furthermore, to our knowledge, there remains an unexplored area of research regarding the effectiveness of the nine BCW intervention functions specifically in the area of precision prevention, as well as the outcomes of combining multiple intervention functions. There is an urgent need for research that systematically evaluates which intervention function is most effective for different populations and tasks. Furthermore, the efficacy of these digital interventions in real-world settings has to be studied by conducting long-term field trials to uncover insights as to whether the interventions lead to improved health and well-being in the target group. This would contribute significantly to the further development and optimisation of precision prevention strategies.

The child-focused intervention implemented only four intervention components of the BCW [ 10 ]. This decision was probably made to avoid overwhelming the young target group with excessive functionality. Such a strategy aligns seamlessly with established findings from the fields of technology acceptance and user experience. In these areas, usability emerges as a critical attribute of any digital health solution. A user-friendly interface significantly increases the likelihood that individuals will engage with the solution. This concept is closely related to ‚effort expectancy‘, a key element of the Unified Theory of Acceptance and Use of Technology [ 17 ]. Furthermore, the principle of usability is recognised as a fundamental human factor, underlining its importance in the design and adoption of technological solutions.

The studies that have been conducted have overlooked a crucial aspect: they have not evaluated or documented the impact of behavioral components implemented in digital health solutions on the effectiveness of the precision prevention intervention. Although a review of three selected case studies prevents us from generalizing these results, based on our experience in this domain, this is still an open research issue. There remains a significant gap in research on the effective integration of different behavioral elements into digital health interventions. In the three case studies, recommendations were disseminated through the digital solution and data were collected through this solution. It is important to note, however, that this data collection was based solely on self-reporting and did not include automated data collection. A key feature of all three solutions was the provision of feedback to users based on their self-reported data. However, there is a noticeable lack of information on how users perceived and accepted this feedback. Furthermore, the studies provided little detail on the visual aspects of these incentivisation strategies, an area that warrants greater attention and exploration.

In the case studies, although educational content was disseminated through digital solutions, it is worth noting that only basic functionality was used for this purpose. This approach underlines an important finding: all cases used a blended learning approach, combining digital and face-to-face education. This hybrid model highlights the potential of technology for realizing this function, the studies used more traditional methods, such as weekly meetings and the use of labeled water bottles. This choice to implement ‚enablement‘ through traditional rather than digital means opens up a discussion about the effectiveness and appropriateness of different intervention methods in different contexts, and suggests a potential area for further exploration in the integration of digital tools in behavioral interventions.

Tailoring is a key component in precision prevention interventions. Although the interventions designed in the selected studies were tailored to the target population, the technology played either no role or a limited role in this tailoring. As exemplified, the digital solution used in the studies [ 10 ] provided predefined content tailored to the user based on their age and nutritional status. In this regard, the technology selected a recommendation from a set of predefined contents based on the manually entered data. These collected data belonged to the “users” dimension, that is only one of the four personalization dimensions identified by Gosetto *et al.* , [ 18 ]. The digital solution used a simple algorithm to decide what needed to be recommended. Tailoring was performed based on an individual's data entered into the system and other relevant factors related to the context in which the behavioral change. More advanced tailoring strategies that, for example, learn about a users‘ preferences as they use the system or detect when a change in behavior has occurred and adapt the system were not implemented. This finding is in line with the results reported by Monteiro-Guerra *et al.* , [ 19 ]. Artificial intelligence (AI) has been used to implement advanced personalization strategies to generate more attractive and encouraging user experiences that motivate them to reach their objectives of maintaining a healthy lifestyle [ 20 ]. As an example, generative AI models such as large language models could be used to automatically generate real-time contents that meet the specific user's preferences, status, and needs [ 21 ]. BCW functions could be combined with AI-based personalization strategies to develop persuasive digital health solutions that enable more effective precision prevention interventions. The use of AI models for precision prevention also requires addressing some relevant aspects such as ethical and privacy issues [ 22 ].

As mentioned before, despite the limited role of technology in tailoring the precision prevention intervention, there is a lack of evidence on the impact of implementing personalization or tailoring strategies in the digital solution on the effectiveness of the intervention. Most of the studies analyzed the effectiveness of the digital intervention, but researchers did not conduct any experiment to assess how the digital tailoring was influencing its effectiveness. This evaluation will have a crucial part in generating new knowledge that allows progress on the state-of-the-art of persuasive health technologies for precision prevention. In this regard, further research should explore how digital tailoring is influencing the effectiveness of the intervention considering relevant factors such as age, gender, digital literacy, etc. In addition, a deeper analysis should assess the impact of each individual tailoring technique on this effectiveness considering some factors such as user's characteristics, context factors, and/or intervention components.

## 5. Conclusions

This study highlights the application and challenges of integrating behavioral components into digital health interventions for precision prevention. Despite this lack of evidence and the limited role of technology in the tailoring of the precision prevention interventions, it should be noted that the overall results were very promising, demonstrating the benefits of digital health in the precision prevention field. A key finding is the urgent need for a framework to effectively tailor behavior components within such solutions. We identify significant evidence gaps in both the tailoring process and the effectiveness of behavior change techniques in precision prevention. Additionally, there is a critical lack of data on the personalisation of behavioral components and a lack of design guidelines for the effective integration of these components, particularly in persuasive technologies. This lack of evidence highlights the need for further research to improve the effectiveness of behavior components in precision prevention strategies.

In conclusion, while our study provides valuable insights, it also highlights the need for more focused studies and guidelines to optimize these interventions for individualized health outcomes. Addressing these gaps will be critical to advancing precision prevention in digital health.
